# Do Molecular Geometries Change Under Vibrational Strong Coupling?

**DOI:** 10.1021/acs.jpclett.4c01810

**Published:** 2024-07-23

**Authors:** Thomas Schnappinger, Markus Kowalewski

**Affiliations:** Department of Physics, Stockholm University, SE-106 91 Stockholm, Sweden

## Abstract

As pioneering experiments have shown, strong coupling between molecular vibrations and light modes in an optical cavity can significantly alter molecular properties and even affect chemical reactivity. However, the current theoretical description is limited and far from complete. To explore the origin of this exciting observation, we investigate how the molecular structure changes under strong light−matter coupling using an ab initio method based on the cavity Born−Oppenheimer Hartree−Fock ansatz. By optimizing H_2_O and H_2_O_2_ resonantly coupled to cavity modes, we study the importance of reorientation and geometric relaxation. In addition, we show that the inclusion of one or two cavity modes can change the observed results. On the basis of our findings, we derive a simple concept to estimate the effect of the cavity interaction on the molecular geometry using the molecular polarizability and the dipole moments.

**Figure:**
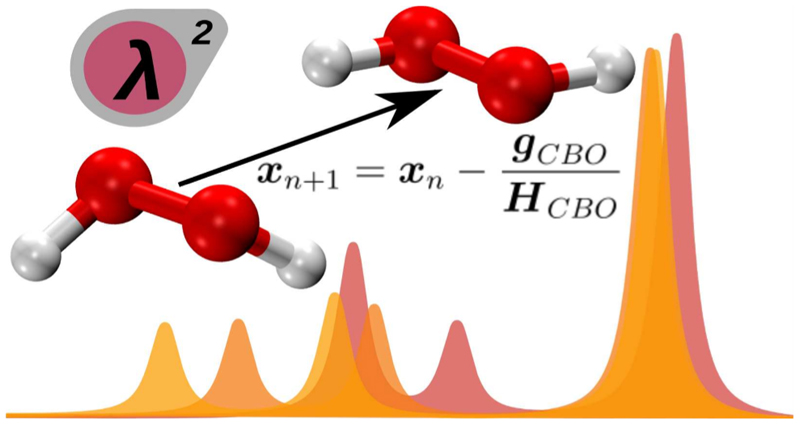

**W**hen molecules are placed in a nonclassical photonic environment present in optical or nanoplasmonic cavities, it is possible to form strong light−matter-coupled hybrid states called polaritons.^1–6^ The control of the photonic environment allows to couple the cavity photon modes to vibrational or electronic transitions in molecules, called vibrational-strong coupling (VSC) or electronic-strong coupling (ESC), respectively. Both types of strong coupling can be an effective tool for modifying molecular properties and offer a possible novel approach to control chemical reactions using optical resonators. The experimental advances reported cover a wide range of applications, from manipulating the selectivity of organic reactions,^7–9^ changing the ionic conductivity of water^10^ to even influencing enzymatic activity.^11,12^ Driven by these experimental advances, considerable efforts have been made to develop theories that elucidate the mechanisms governing polaritonic chemistry. Even so, the current theoretical description is limited and far from complete. However, in recent years, a substantial number of studies using different theoretical approaches have proposed that a variety of additional reactions can be enhanced, inhibited, or controlled.^13–25^ In particular, the combination of electronic structure methods and quantum electrodynamics^26–34^ has significantly improved theoretical understanding and will hopefully help close the existing gaps between theory and experiment. Most of these studies model cavity-induced electronic structure changes using a single molecule coupled to a single-cavity mode in the strong-coupling limit. In almost every example, both a fixed orientation relative to the polarization axes of the cavity mode and fixed molecular geometries are assumed, despite the fact that the coupling strengths used are quite large. Recently, first studies^35–37^ have highlighted the importance of orientation with respect to the cavity polarization mode.

In this work, we use the cavity Born−Oppenheimer Hartree−Fock (CBO-HF) ansatz^31^ together with analytical gradients^38^ to optimize H_2_O and H_2_O_2_ resonantly coupled to one and two cavity photon modes. The CBO-HF ansatz is capable of describing the electronic ground state of single molecules, as well as an ensemble of molecules coupled to an optical cavity under VSCs conditions.^31,33,39^ By optimizing the geometries in the laboratory frame together with the polarization vectors of the cavity, we are able to study both the orientation and the relaxation of the internal coordinates of the molecules induced by the interaction with the cavity photon modes. Furthermore, we compute vibro-polaritonic IR spectra^38^ within the harmonic approximation, which allow us to verify the structures found as real minima and to analyze in detail the polaritonic states formed. As will become clear in the remainder of this article, without a physical mechanism to fix the orientation, molecules inside an optical cavity will orient and change their geometry depending on the coupling strength. Furthermore, these observed effects will vary if one- or two-cavity orthogonal photon modes are included in the simulation. The main results of this work indicate that some theoretical studies in the literature may overestimate the cavity-induced effects on the ground-state chemistry. Finally, we establish a useful and straightforward connection between the molecular polarizability and dipole moment and the expected reorientation and relaxation of a molecule coupled to an optical cavity.

Starting from the nonrelativistic Pauli−Fierz Hamiltonian in the length gauge representation,^40–44^ we apply the cavity Born−Oppenheimer approximation (CBOA)^45–48^ to simulate molecules interacting with the confined electromagnetic field in an optical cavity under VSC conditions. By making use of a generalized Born−Huang expansion^44,49^ the cavity modes are grouped with the nuclei and the resulting electronic subsystem can be solved in an ab initio manner.^31,33,50^ Atomic units (*ħ* = 4*πε*_0_ = *m*_*e*_ = 1) are used in the following unless otherwise noted, and bold symbols denote vectors. The electronic CBOA Hamiltonian for multiple cavity modes takes the form of

(1) $${\hat{H}}_{\text{CBO}}={\hat{H}}_{\text{el}}+\sum_{m}^{N_{M}}\frac{1}{2}\omega_{m}^{2}q_{m}^{2}-\omega_{m}q_{m}\left(\lambda_{m}\cdot \hat{\mu }\right)+\frac{1}{2}{\left(\lambda_{m}\cdot \hat{\mu }\right)}^{2}$$

where $\hat{\mu }$ represents the molecular dipole operator, which is defined by the operators of the *N*_el_ electron coordinates $\hat{r}$ and the classic coordinates ***R*** of the *N*_Nuc_ nuclei. *Ĥ*_el_ is the Hamiltonian for the field-free many-electron system. Each of the *N*_*m*_ cavity modes contributes three additional terms to the electronic Hamiltonian. The first term is a harmonic potential introduced by the photon displacement field, with the classic photon displacement coordinate *q*_*m*_ and *ω*_*m*_ being the frequency of the cavity mode. The second term describes the dipole coupling between the molecular system and the photon displacement field, which is characterized by the coupling strength ***λ***_*m*_. The last term is the dipole self-energy (DSE) operator,^51–53^ which is an energy contribution that describes the self-polarization of the molecule-cavity system. The cavity mode-specific coupling parameter ***λ***_*m*_ for a cavity with effective mode volume *V*_*m*_ is defined as follows:

(2) $$\lambda_{m}=e_{m}\lambda_{m}=e_{m}\sqrt{\frac{4\pi }{V_{m}}}$$

The unit vector ***e***_*m*_ denotes the polarization axis of the cavity mode *m*.

The many-electron problem described by eq 1 can be solved using the CBO-HF approach.^31^ The resulting energy *E*_CBO_ is a function of the nuclear coordinates ***R*** and the photon displacement coordinates ***q***, represented as a vector grouping all *q*_*m*_:

(3) $$\begin{array}{l} E_{\text{CBO}}=\langle {\hat{H}}_{\text{CBO}}\rangle (\text{R},\text{q})\\ \;=\langle {\hat{H}}_{\text{el}}\rangle (\text{R})+\overset{N_{M}}{\underset{m}{\sum }}-\omega_{m}q_{m}\langle \lambda_{m}\cdot \hat{\text{μ}}\rangle (\text{R})+\frac{1}{2}\langle {\left(\lambda_{m}\cdot \hat{\text{μ}}\right)}^{2}\rangle (\text{R})+\frac{1}{2}\omega_{m}^{2}q_{m}^{2} \end{array}$$

The first derivative of the energy *E*_CBO_ with respect to the nuclear and photon displacement coordinates can be calculated analytically^38^ and defines the CBO-HF gradient ***g***_CBO_ as a (3*N*_A_ + *N*_*M*_) vector, where *N*_A_ is the number of atoms in the molecule.

(4) $$\text{g}=\nabla E_{\text{CBO}}$$

Based on the analytic gradients, the CBO−Hessian matrix ***H*** of size (3*N*_A_ + *N*_*M*_)(3*N*_A_ + *N*_*M*_) is accessible via finite differences.^38^

(5) $$H_{ij}=\nabla_{i}g_{j}\approx \frac{g_{j}\left(x_{i}+\text{Δ}\right)-g_{j}\left(x_{i}-\text{Δ}\right)}{2\text{Δ}}$$

with *x*_*i*_ being a nuclear or displacement coordinate. The molecular polarizability tensor *α* at the CBO-HF level is calculated as the first derivative of the CBO-HF dipole moment vector with respect to a small external field, also using finite differences. Both the analytic gradient and the numerical Hessian can be used to optimize molecules coupled to cavity photon modes. In this manuscript we use the Broyden−Fletcher−Goldfarb−Shanno (BFGS) algorithm:

(6) $$x_{n+1}=x_{n}-{\tilde{H}}^{-1}g_{\text{CBO}}$$

where *x* is the combined nuclear and displacement coordinate vector (3*N*_A_ + *N*_*M*_) and ***g***_CBO_ the gradient vector. The approximate Hessian matrix $\tilde{\text{H}}$ at each point *n* is updated with the approximate Hessian matrix at step *n* − 1 according to

(7) $${\tilde{H}}_{n}={\tilde{H}}_{n-1}+\frac{\text{Δ}g\cdot \text{Δ}g^{T}}{\text{Δ}g^{T}\cdot \text{Δ}x}-\frac{{\tilde{H}}_{n-1}\cdot \text{Δ}x\cdot \text{Δ}x^{T}{\tilde{H}}_{n-1}}{\text{Δ}x^{T}\cdot {\tilde{H}}_{n-1}\cdot \text{Δ}x}$$

A detailed benchmark of the implemented BFGS algorithm against the Steepest Descent method and the Newton−Raphson method can be found in the Supporting Information

The BFGS algorithm, the necessary analytical gradients and the numerical Hessian for the CBO-HF ansatz have been implemented in the Psi4NumPy environment,^54^ which is an extension of the PSI4^55^ electronic structure package. All calculations were performed using the aug-cc-pVDZ basis set^56^ and all geometries were preoptimized at the Hartree−Fock level of theory. In all CBO-HF calculations performed in this work, we consider a lossless cavity. The coupling strength *λ*_*m*_ is chosen between 0.015 au and 0.150 au to assess the medium and strong coupling situation in the case of a single molecule. We emphasize that parts of the light-matter coupling used here are significantly larger than what can presently be achieved in experiments. For example, *λ*_*m*_ = 0.100 au corresponds to an effective mode volume of less than 0.2 nm^3^, which is less than the typical mode volumes of approximately 10.0 nm^3^ that can be achieved in plasmonic cavities.^1,57^

The CBO-HF energy and gradients depend on the relative orientation of the molecule and the polarization axes of the cavity. For this reason, the internal coordinate system traditionally used to optimize molecular geometries is a poor choice because it neglects spatial orientation. Therefore, all geometry optimizations in this work were performed using Cartesian coordinates. For the first step of the BFGS optimization, the exact Hessian matrix was computed, and to improve convergence for the H_2_O optimization, the exact Hessian was recomputed after every 10th step. The maximum component and the root-mean-square deviation of the gradient and the displacement vector are used as convergence criteria. For the gradient, both must be less than 10^−5^ au and for the displacement vector, both must be less than 10^−4^ au. The semiclassical harmonic approximation^38^ was used to determine the normal modes and frequencies of the optimized coupled cavity-molecular systems. All optimized structures discussed in this manuscript are real minima, since they have no imaginary frequencies.

As a first example, we optimize a H_2_O molecule coupled to one photon mode of an optical cavity. The cavity frequency *ω*_*m*_ is resonant with the bending mode, which has a field-free vibrational frequency of 1744 cm^−1^, and the cavity polarization axis ***e*** is aligned with the *x* axis of the laboratory frame. Figure 1 shows the optimized parameters for the coupled molecular cavity system as a function of the coupling strength *λ*_*m*_ and the corresponding vibro-polaritonic IR spectra in the region of the H_2_O bending mode.

For the optimized single-mode cavity−H_2_O system, the magnitude of the dipole moment |⟨*μ*⟩|, shown in Figure 1a, is only slightly reduced with increasing *λ*_*m*_. This rather small change is consistent with the observed small decrease in both the OH bond length (Figure 1c) and bond angle (Figure 1d), even at high coupling strengths. The only system parameter significantly affected by VSC is the relative orientation of the molecular dipole moment and the polarization axis of the cavity mode visualized as the angle *ϕ* between them in Figure 1b. For the arbitrary chosen initial configuration, *ϕ* has a value of about 55° (dashed dotted line), which changes to exactly 90° after optimization, regardless of the coupling strength. The interaction with the photon field leads to an orientation of the molecule such that not only the dipole moment but also the molecular plan is orthogonal to the polarization axis. The relevant parts of the vibro-polaritonic IR spectra for the optimized water-cavity system are shown in Figure 1e,f for different coupling strengths. Given the orientation of the dipole moment perpendicular to the cavity polarization axis, one would expect no hybrid photonic states to be formed because of the lack of dipole-cavity interaction. This assumption holds for lower coupling strengths below 0.062 au, shown in Figure 1e. Within the used broadening of 10 cm^−1^, only a single peak is observed, almost unshifted to the field-free value (black dashed line). For *λ*_*m*_ = 0.062 au a small shoulder appears at a slightly lower frequency, the bright purple line in Figure 1 e). If the coupling is further increased, as shown in Figure 1f, this shoulder becomes a separate peak with increasing intensities and shifts to lower frequencies with increasing *λ*_*m*_. As introduced in our early work,^38^ the normal mode component that describes the change in the classical photon displacement field is a measure of how much photon character the corresponding transition has. The main peak in all shown vibro-polaritonic IR spectra has no photonic character and can be described as a pure molecular transition corresponding to the bending mode. The smaller peak at higher coupling strengths is predominantly photonic, and we do not see the formation of a typical hybrid matter−photon lower polariton (LP) state and upper polariton (UP) state here. This can be explained by a rotational motion of the H_2_O molecule. Due to the confinement of the cavity, this motion is no longer a free rotation. This motion induces a dipole moment parallel to the cavity polarization axis, which leads to a coupling with the photon mode. Due to this coupling, the photonic transition gains intensity and shifts to lower frequencies. More details and a thorough analysis of the photonic characters of all relevant transitions can be found in the Supporting Information.

Next, we discuss the optimization results for H_2_O coupled to two cavity modes of orthogonal polarization with the same frequency, effectively modeling a Fabry−Pérot-like setup. In practice, we use the same cavity frequency and coupling strength for both modes and take the same values as for the single-mode case. The polarization axes are aligned with the *x* axis (***e***_1_) and the *y* axis (***e***_2_) of the laboratory frame. The optimized parameters as a function of the coupling strength *λ*_*m*_ and the corresponding vibro-polaritonic IR spectra in the region of the H_2_O bending mode are shown in Figure 2.

When the optimized parameters are compared for the single-mode case and the two-mode case, some similarities but also distinct differences are observed. The change in magnitude of the dipole moment, shown in Figure 2a, is nearly 1 order of magnitude smaller for the two-mode optimization. In contrast, the OH bond length (Figure 2c) and the bond angle (Figure 2d) are more strongly influenced by the coupling to two cavity modes. In particular, the change in the bond angle is significantly larger compared to the case of a single mode, although it increases only by 1.5° for the highest coupling strength. Again, the most significant changes due to optimization are the relative orientations of the molecular dipole moment with respect to the cavity-mode polarization axes. The corresponding angles *ϕ* for ***e***_1_ and ***e***_2_ are shown in Figure 2b. Starting from a configuration where both ***e***_1_ and ***e***_2_ are in the molecular plane but not aligned with the dipole moment, the optimization reorients the molecule independently of the coupling strength, so that ***e***_1_ is parallel to the dipole moment, while ***e***_2_ is orthogonal to the dipole moment and the molecular plane. The corresponding vibro-polaritonic IR spectra for the optimized water-cavity system are shown in Figure 2e,f for different coupling strengths. Already, for the lowest coupling strength, *λ*_*m*_ = 0.015 au in Figure 2e, a splitting into a LP transition and a UP transition is observed. The corresponding normal modes clearly show a hybridization of the vibrational transition and the cavity photon mode with the polarization axis ***e***_1_. As *λ*_*m*_ increases, the Rabi splitting between the LP and UP transitions increases and becomes more asymmetric, consistent with our previous work.^38,39^ At the same time, a third weaker peak between the LP and UP transitions becomes visible. Similarly to the single-mode case, this peak is due to the coupling between a specific rotational degree of freedom and the photon mode with the polarization axis ***e***_2_. Due to this coupling, the photonic transition gains intensity and shifts to low frequencies with increasing coupling strength. More information on the photonic characters of all relevant transitions can be found in the Supporting Information

To gain more insight into the underlying driving force for the energetically favorable orientation, we discuss how VSC modifies the polarizability *α* and the DSE contribution. The principal components of the polarizability tensor *α* and the change in the DSE contribution are shown in Figure 3 as a function of the coupling strength for both optimized H_2_O−cavity systems.

In the single-mode case shown in Figure 3a and the two-mode case shown in Figure 3b, all three principal components of *α* are reduced with increasing coupling strength or, in other words, the interaction with the photon mode contracts the electronic density. Therefore, it is logical that the components aligned with the cavity polarization axes (highlighted in bold) are the most affected. These components already have the smallest value in the field-free case, and the optimization reorients the molecule so that these components align with the cavity polarization axes. For H_2_O these are the component orthogonal to the molecular plane corresponding to the green lines in Figure 3a,b and the parallel component to the molecular dipole moment corresponding to the orange lines in Figure 3a,b. As shown in Figure 3c, the observed reorientation also minimizes the DSE contribution to the coupled cavity−molecule system. This is in agreement with our previous work^31^ and a recent study by Liebenthal and DePrince.^37^ In general, reorientation can be rationalized as an effective way to reduce molecular polarizability along the cavity polarization axes and thus minimizing the DSE contribution.

All results presented for the optimization of H_2_O resonantly coupled to a single or two orthogonal cavity photon modes show that, without restriction of the rotational degrees of freedom, the cavity interaction leads to a reorientation of the molecule. The observed reorientation is visualized in Figure 4 for the case of a single cavity mode (a) and two cavity modes (b). This rotational motion occurs for all coupling strengths and is more pronounced than changes in the internal coordinates of H_2_O. For higher coupling strengths, the rotational effects even become visible in the vibro-polaritonic IR spectra.

The optimization of a H_2_O_2_ molecule coupled to a single photon mode and two to orthogonal photon modes serves as the second example. The cavity is resonant with the asymmetric bending mode of 1491 cm^−1^, and the cavity polarization axis *e* in the case of a single mode is aligned with the molecular dipole moment, which corresponds to the *z* axis of the laboratory frame defining the *C*_2_ symmetry axis of H_2_O_2_. The optimized parameters for the coupled molecular cavity system as a function of the coupling strength *λ*_*m*_ and the relevant part of the corresponding vibro-polaritonic IR spectra are shown in Figure 5.

Regardless of the coupling strength used, the dipole moment of H_2_O_2_ remains aligned with the polarization axis of the single cavity mode, as shown in Figure 5b. However, its size decreases significantly with increasing coupling up to the situation where the dipole moment is zero at *λ*_*m*_ = 0.150 au (see Figure 5a). This large change in the dipole moment with increasing coupling strength is induced by an increase in the dihedral angle, leading to a planarization of H_2_O_2_ (see orange line Figure 5 d)). For *λ*_*m*_ = 0.150 au the H_2_O_2_ molecule becomes completely planar in a *trans* configuration, which results in a zero dipole moment. Note that for this *trans* structure, the cavity polarization axis is orthogonal with respect to the molecular plane, very similar to the H_2_O case. In contrast, the bond lengths and the averaged bond angle shown in Figure 5c and d, respectively, remain nearly constant for increasing coupling strengths. The observed change in the molecular geometry due to the cavity interaction can be clearly seen in the corresponding vibro-polaritonic IR spectra, shown in Figure 5e,f for different coupling strengths. Details about the photonic characters of all relevant transitions can be found in the Supporting Information For smaller values of *λ*_*m*_, as depicted in Figure 5e, there is a clear splitting into a LP transition and a UP transition, which increases with increasing coupling strength. If *λ*_*m*_ is further increased, the changes in dipole moment and dihedral angle are more pronounced, which changes the vibro-polaritonic IR spectra, see Figure 5f. These spectra are characterized by a single peak that is a pure molecular transition but is red-shifted relative to the field-free asymmetric bending mode. For a coupling strength below 0.13 au a small shoulder a slightly lower frequency is present. This signal has a photonic character and is visible due to the weak coupling of the photon mode with the twisting mode in H_2_O_2_ at 424 cm^−1^. This mode is the most intense transition in the field-free spectrum and is characterized by a change in the dihedral angle of H_2_O_2_. Similarly to the rotational motion observed in H_2_O, this twisting mode induces a dipole moment parallel to the polarization axis of the cavity. However, as the coupling increases further and H_2_O_2_ becomes more planar, this coupling weakens, and only the single molecular peak remains visible.

To optimize the H_2_O_2_ molecule coupled to a two mode cavity, we add a second orthogonal cavity mode with the same frequency and coupling strength. The two polarization axes are aligned with the *z* axis (***e***_1_) and the *x* axis (***e***_2_) of the laboratory frame. The optimized parameters as a function of the coupling strength *λ*_*m*_ and the relevant part of the vibro-polaritonic IR spectra are shown in Figure 6.

Optimization of H_2_O_2_ coupled to a two-mode cavity setup leads to similar but significantly weaker changes than in the case of coupling to a single cavity mode. The dipole moment is reduced with increasing coupling strength, see Figure 6a, while the dihedral angle is simultaneously increased, see Figure 6d, orange line. The other internal coordinates of H_2_O_2_, shown in Figure 2c,d, are slightly more affected in the two-mode case, but still in a rather insignificant way. Similarly to the single-mode coupling, we do not observe any reorientation of the H_2_O_2_ dipole moment with respect to the polarization axes for the two-mode case due to the chosen orientation of the initial geometry. The dipole moment is parallel to the polarization axis ***e***_1_ and therefore orthogonal to the other, as visualized in Figure 6b. Next, we discuss the vibro-polaritonic IR spectra of the optimized H_2_O_2_ molecule coupled to a two-mode cavity. Figure 6e shows spectra for smaller coupling strengths, which can be roughly divided into two groups. When *λ*_*m*_ is less than 0.05 au, a weak Rabi splitting is observed. The LP transition and the UP transition are formed by the asymmetric bending mode and the cavity photon mode with the polarization axis ***e***_1_. This “standard” pair of LP and UP is present in all spectra, and the Rabi splitting increases with increasing coupling strength, see Figure 6e,f. But similar to the case of H_2_O coupled with two cavity modes, a third weaker signal is present first as a shoulder (*λ*_*m*_ = 0.05 au, Figure 6e) and later as a distinct peak, as shown in Figure 6f. This weaker “middle” signal is mostly photonic and is characterized by the cavity photon mode with the polarization axis ***e***_2_. In line with the single-mode case, this transition is due to a weak coupling to the twisting mode in H_2_O_2_ for higher coupling strengths. A visualization of the photonic characters of all relevant transitions can be found in the Supporting Information

As discussed for the optimization of H_2_O, we also want to understand the observed changes in H_2_O_2_ due to the cavity interaction by analyzing the polarizability *α* and the DSE contribution. The principal components of the polarizability tensor *α* and the change in the DSE contribution are shown in Figure 7 as a function of the coupling strength for both optimized H_2_O_2_-cavity systems.

Consistent with the H_2_O results shown in Figure 3, in both the single-mode case and the two-mode case, all three principal components of the polarizability *α* decrease with increasing coupling strength, see Figure 7a,b. The components aligned with the cavity polarization axes, highlighted in bold, are more strongly reduced by the cavity interaction, while especially for the single-mode case (Figure 7a), the other two components remain almost unchanged. We do not see reorientation of H_2_O_2_ for the cavity polarization axes studied in the single-mode and two-mode cases, since for the chosen molecular orientation the axes are already aligned with the smallest polarizability components *α*^I^ and *α*^II^. Consequently, the observed reduction in polarizability is achieved only by geometrical changes and the direct response of the electronic structure to the cavity photon field. These geometrical changes and the electronic response also minimizes the DSE contribution. The change in *E*_DSE_ is plotted as a function of *λ*_*m*_ in Figure 7c. Interestingly, the energy change is much larger for the single-mode case (green line) compared to the two-mode case (orange line), although the change in *α* is comparable. To explain this, we have to consider that the total DSE contribution can be separated into a one-electron and two-electron part.^31,33,39^ The behavior of the one-electron contribution, which is usually the larger part, can be estimated by the polarizability, while the two-electron contribution is defined by the product of the dipole moment with itself. Since in the single-mode case H_2_O_2_ planarizes with increasing coupling strength, its dipole moment decreases and is minimal in the fully planar *trans* configuration. Consequently, the two-electron part of the DSE becomes smaller and finally is exactly zero. In the single-mode case, the change in DSE reflects both the decrease in polarizability and the decrease in the dipole moment. The present result for the optimization of H_2_O and H_2_O_2_ coupled to an optical cavity clearly shows that not only the dipole moment of the molecule is important, but also the polarizability is a decisive factor in determining the influence of the molecule-cavity interaction.

To conclude, we studied the importance of geometry relaxation and relative orientation in the context of molecules coupled to the photon modes of optical cavities. Both effects are currently neglected in most computational studies in the field of polaritonic chemistry. As two illustrative examples, we have optimized H_2_O and H_2_O_2_ resonantly coupled to one or two cavity photon modes. For the case of H_2_O, the predominant effect observed during the optimization processes is a rotation that leads to a reorientation of the molecule with respect to the photon modes, independent of the coupling strength studied. Surprisingly, we could even observe an effect of the restricted rotational degrees of freedom on the vibro-polaritonic IR spectrum for larger coupling strengths. These results clearly show that rotational motion is no longer an unrestricted degree of freedom in a cavity molecular system that can be neglected. In contrast, for the optimization of H_2_O_2_, no rotational motion occurred for the chosen initial conditions (orientation of the molecule and polarization axes of the cavity), and geometric relaxation is more important. Consequently, the interpretation of computational studies based on fixed molecular structures and fixed orientation should be viewed with caution. Comparing the optimizations of H_2_O_2_ coupled to one- and two-cavity modes, we want to highlight that the results have some similarities, but also show significant differences. The more realistic case with two cavity modes predicts smaller changes in the molecular structure, while the single-mode case over-emphasizes them, even leading to a planar structure of H_2_O_2_.

In agreement with recent studies,^31,37^ we can confirm the minimization of the DSE contribution as the driving force for both orientation and geometric relaxation. The DSE has been shown^31,51–53^ to be strictly necessary to obtain a finite polarization and a bounded solution. However, despite its importance, DSE is still a rather abstract idea, so we derived a more accessible concept to estimate the effect of cavity interaction on molecular geometry. We are able to explain the observed rotation of the cavity-coupled molecule by determining the main components of the polarizability tensor *α*. Without fixing its orientation, the molecule will reorient so that the cavity mode polarization axes align with the smallest components of the polarizability. With respect to geometric relaxation, we observed two effects: First, it aims to reduce the polarizability of the molecule and, second, it also reduces the dipole moment itself. The evaluation of both the dipole moment and the polarizability is likely to be a useful and simple tool to evaluate the possibility of influencing molecules and chemical reactions by strong light-matter interaction inside an optical cavity. Exploring changes in orientation and geometries in molecular ensembles is a logical next step.

## Supplementary Material

Supplementary Material The Supporting Information is available free of charge at https://pubs.acs.org/doi/10.1021/acs.jpclett.4c01810.
Detailed analysis of vibro-polaritonic normal modes, a benchmark of different optimization methods and parameters, and all optimized geometries (PDF)

## Figures and Tables

**Figure 1:**
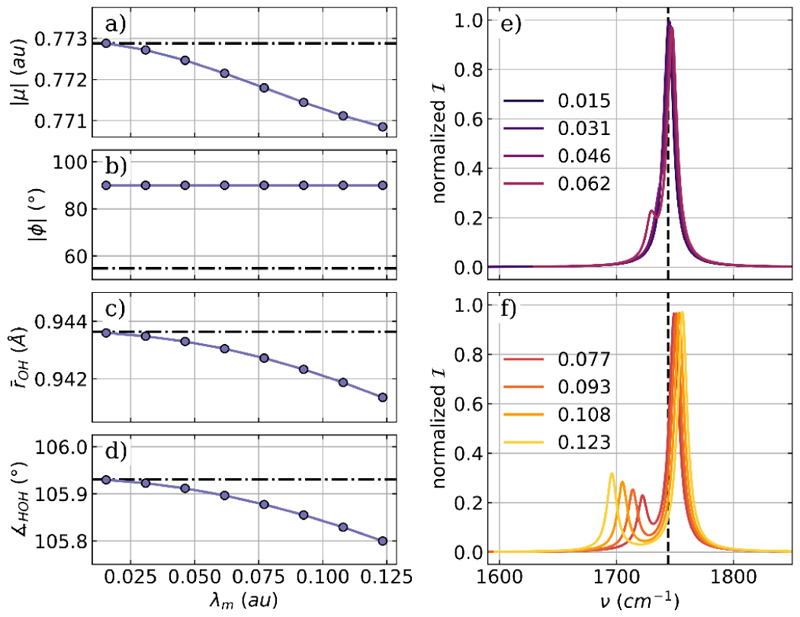
Optimized parameters of a H_2_O molecule coupled to a single photon mode of an optical cavity as a function of the coupling strength *λ*_*m*_. (a) Magnitude of the dipole moment |*μ*|, (b) the angle *ϕ* between the polarization axis of the cavity and the dipole moment, (c) averaged OH bond length, and (d) bond angle. The dashed-dotted lines in (a)−(d) indicate the initial values. Relevant parts of the vibro-polaritonic IR spectra for different coupling strengths (color-coded) are shown in (e) and (f). The cavity frequency *ω*_*m*_ is 1744 cm^−1^, shown as a black dashed line in (e) and (f). The cavity coupling *λ*_*m*_ increases from 0.015 au to 0.123 au, and the cavity polarization axis is ***e*** = (1,0,0).

**Figure 2:**
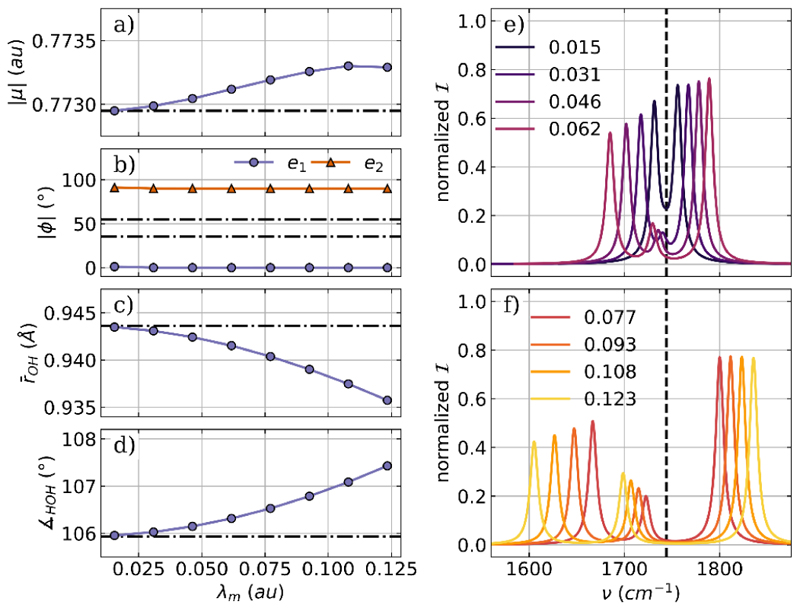
Optimized parameters of a H_2_O molecule coupled to two orthogonal cavity photon modes as a function of the coupling strength *λ*_*m*_. (a) Magnitude of the dipole moment |*μ*|, (b) the two angle *ϕ* between the polarization axes of the cavity and the dipole moment of the molecule, (c) averaged OH bond length, and (d) the bond angle. The dashed-dotted lines indicate the initial values in (a)−(d). The relevant part of the vibro-polaritonic IR spectra for different coupling strengths (color-coded) is shown in (e) and (f). The cavity frequency *ω*_*m*_ is 1744 cm^−1^ shown as a black dashed line in (e) and (f). The cavity coupling *λ*_*m*_ increases from 0.015 au to 0.123 au, and the cavity polarization axes are ***e***_1_ = (1,0,0) and ***e***_2_ = (0,1,0).

**Figure 3:**
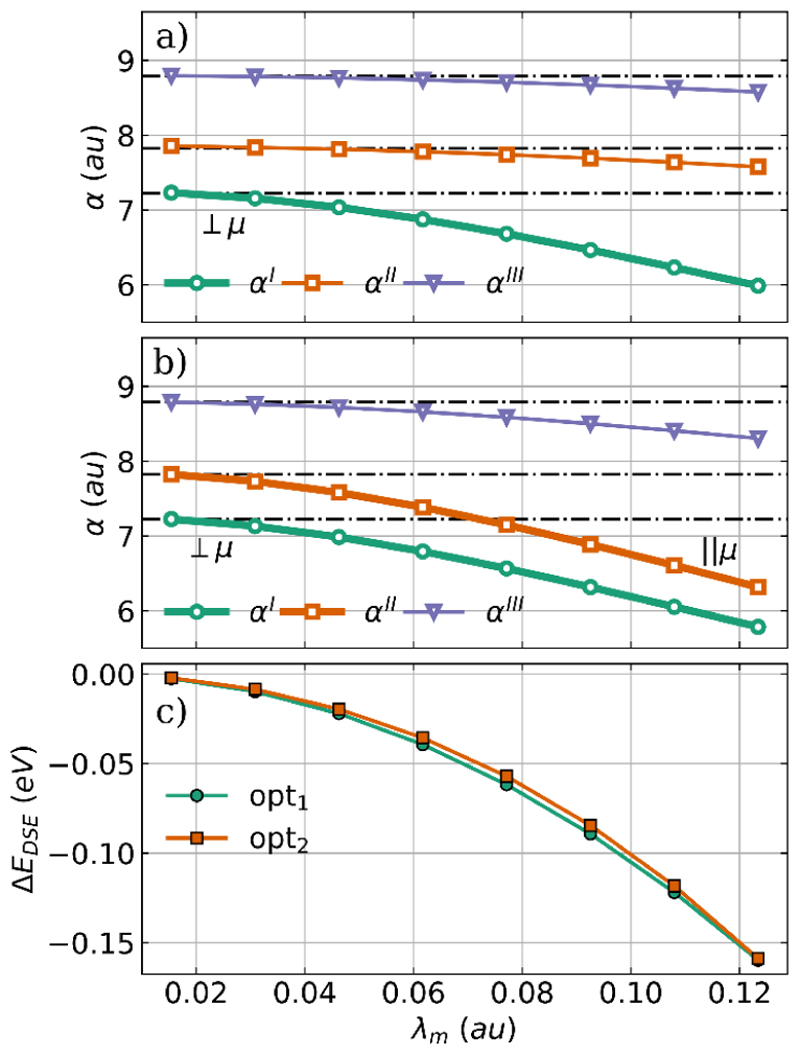
Principal components of the polarizability tensor *α* as a function of the coupling strength *λ*_*m*_ for optimized H_2_O structures coupled to (a) a single cavity mode and to (b) two orthogonal cavity modes. The bold line indicates the component aligned with the cavity polarization axes, and the black dashed dotted lines represent the field-free principal components of the polarizability. (c) Change in DSE contribution as a function of the coupling strength *λ*_*m*_ compared to the initial H_2_O geometry for the single-mode case (green) and the two-mode case (orange). The cavity frequency *ω*_*m*_ is 1744 cm^−1^.

**Figure 4:**
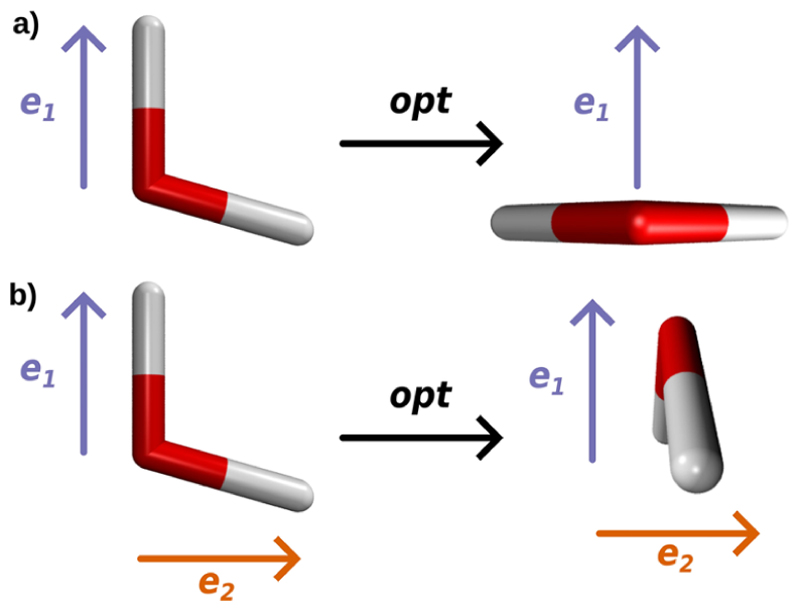
Orientation of the H_2_O molecule with respect to the cavity polarization directions before and after the optimization procedure including a single cavity mode (a) and two cavity modes (b).

**Figure 5:**
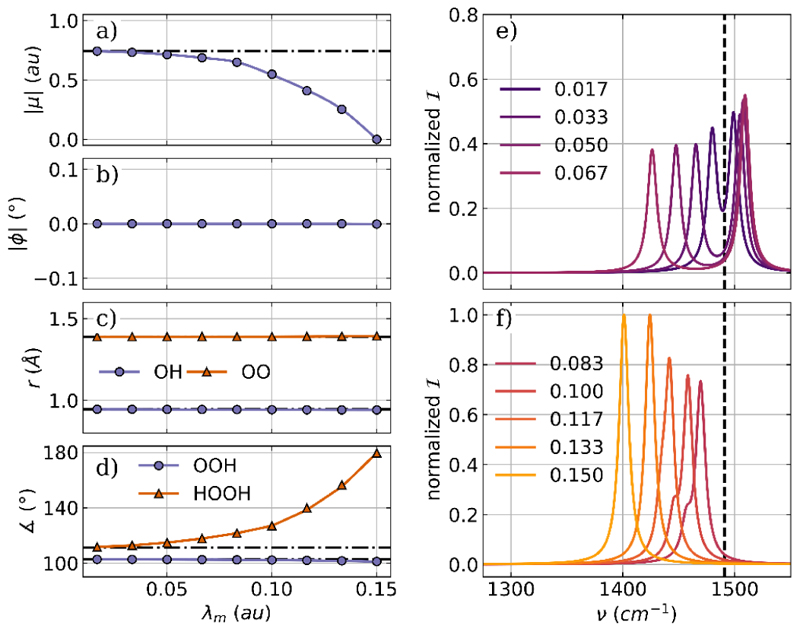
Optimized parameters of a H_2_O_2_ molecule coupled to a single photon mode of an optical cavity as a function of the coupling strength *λ*_*m*_. (a) Magnitude of the dipole moment |*μ*|, (b) the angle *ϕ* between the polarization axis of the cavity and the dipole moment of the molecule, (c) averaged OH bond length (purple) and OO bond length (orange), and (d) the averaged bond angle (purple) as well as the dihedral angle (orange). The dashed-dotted lines in (a)−(d) indicate the initial values. Relevant parts of the vibro-polaritonic IR spectra for different coupling strengths (color-coded) are shown in (e) and (f). The cavity frequency *ω*_*m*_ is 1491 cm^−1^, shown as a black dashed line in (e) and (f). The cavity coupling *λ*_*m*_ increases from 0.017 au to 0.150 au, and the cavity polarization axis is ***e*** = (0,0,1).

**Figure 6:**
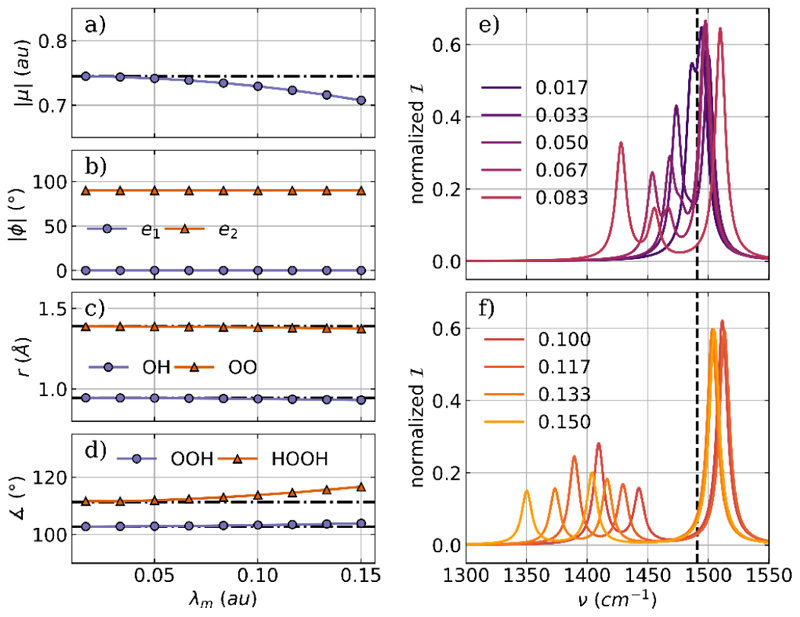
Optimized parameters of a H_2_O_2_ molecule coupled to two orthogonal cavity photon modes as a function of the coupling strength *λ*_*m*_. (a) Magnitude of the dipole moment |*μ*|, (b) the angle *ϕ* between the cavity polarization axes and the dipole moment of the molecule, (c) averaged OH bond length (purple) and OO bond length (orange), and (d) the averaged bond angle (purple) as well as the dihedral angle (orange). The dashed-dotted lines in (a)−(d) indicate the initial values. Relevant parts of the vibro-polaritonic IR spectra for different coupling strengths (color-coded) are shown in (e) and (f). The cavity frequency *ω*_*m*_ is 1491 cm^−1^, shown as a black dashed line in (e) and (f). The cavity coupling *λ*_*m*_ increases from 0.017 au to 0.150 au, and the cavity polarization axes are ***e***_1_ = (0,0,1) and ***e***_2_ = (1,0,0).

**Figure 7:**
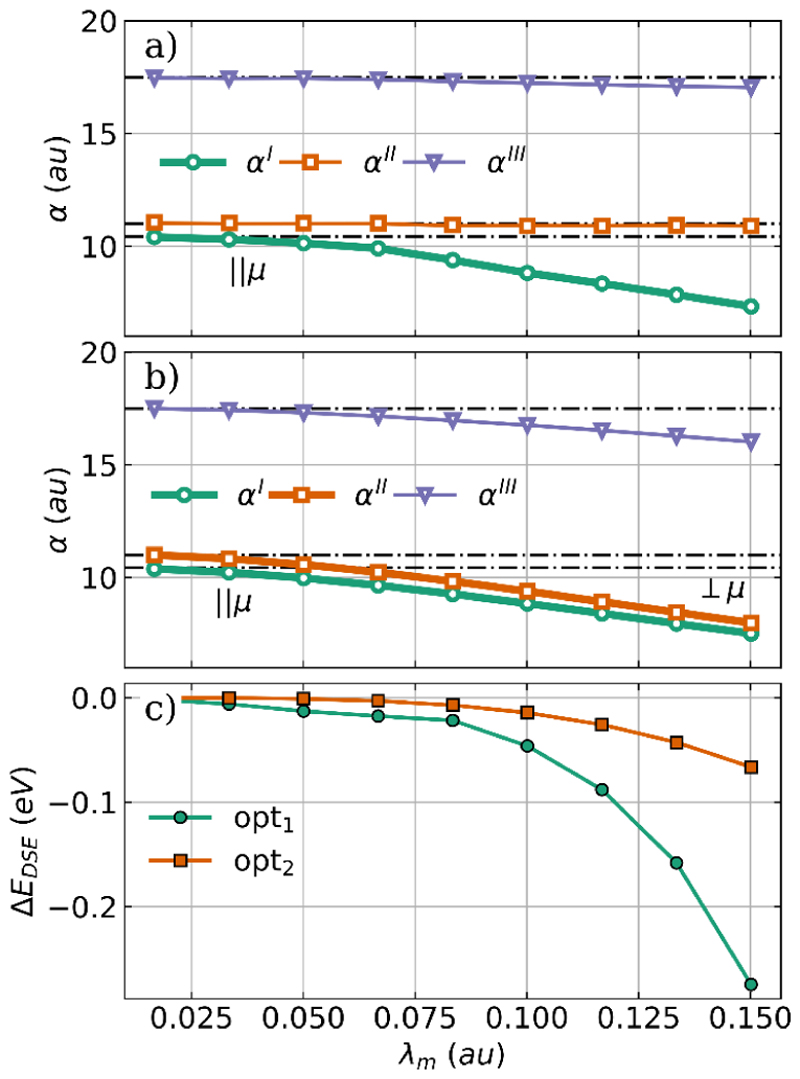
Principal components of the polarizability *α* as a function of the coupling strength *λ*_*m*_ for optimized H_2_O_2_ structures coupled to a single cavity mode (a) and to two cavity modes (b). The bold line indicates the component aligned with the cavity polarization axes, and the black dashed dotted lines represent the field-free principal components of the polarizability. (c) Change in DSE contribution as a function of the coupling strength *λ*_*m*_ compared to the initial H_2_O_2_ geometry for the single-mode case (green) and the two-mode case (orange). The cavity frequency *ω*_*m*_ is resonant with the bending mode (1491 cm^−1^).

## Data Availability

All data underlying this study are available from the corresponding author upon reasonable request.
