# Supplementary material for: Do Molecular Geometries Change Under Vibrational Strong Coupling?
Source: J Phys Chem Lett. Author manuscript; Available in PMC 2024 Aug 9. (PMC11299175; doi:10.1021/acs.jpclett.4c01810)
Supplement: Supplementary Material — The Supporting Information is available free of charge at https://pubs.acs.org/doi/10.1021/acs.jpclett.4c01810. Detailed analysis of vibro-polaritonic normal modes, a benchmark of different optimization methods and parameters, and all optimized geometries (PDF) [file EMS197950-supplement-Supplementary_Material.pdf]

Supporting Information: Do Molecular Geometries Change Under Vibrational Strong Coupling?

Thomas Schnappinger* and Markus Kowalewski*

Department of Physics, Stockholm University, AlbaNova University Center, SE-106 91 Stockholm, Sweden

E-mail: thomas.schnappinger@fysik.su.se; markus.kowalewski@fysik.su.se

Contents

S1 Vibro-Polaritonic Normal Modes Analysis **2**

S2 Benchmark Optimization Methods and Parameters **5**

S3 Optimized geometries **10**

S3.1 Optimized H_2O coupled to a single cavity mode 10

S3.2 Optimized H_2O coupled to two orthogonal cavity modes 12

S3.3 Optimized H_2O_2 coupled to a single cavity mode 14

S3.4 Optimized H_2O_2 coupled to two orthogonal cavity modes 16

References **18**

S1 Vibro-Polaritonic Normal Modes Analysis

The general concept of performing a normal mode analysis in the cavity Born-Oppenheimer approximation (CBOA) was introduced in our previous work,[1] and the reader is referred to the paper for details of the theory. For convenience, the main ideas are summarized in the following. The harmonic approximation gives access to the normal modes $\boldsymbol{a}_i$. In the CBOA the normal mode vectors have terms a_c describing the change in the classical photon displacement field coordinates q_m. The value of $|a_c|^2$ for a given normal mode is a measure of how strongly the corresponding vibrational transition interacts with the photon field. For an uncoupled light-matter system, a pure molecular transition is characterized by a $|a_c|^2$ value of zero, whereas the bare photon mode has a value of one. Note that due to the length gauge description q_m and the corresponding value a_c are no longer a pure photonic quantity if light and matter are coupled.[2–4] However, $|a_c|^2$ can still be used as a probe to identify how photonic the corresponding vibrational transition is. The information obtained is comparable to the coefficients in the Hopfield models.[5]

In the following, we use the $|a_c|^2$ values to characterize all relevant transitions in the vibro-polaritonic IR spectra of all optimized structures. For the case of the coupled H_2O-cavity system, we also discuss the signal intensities $\mathcal{I}$ in the harmonic approximation. The intensities are calculated as the projection of the dipole moment gradient on the normal mode vectors $\boldsymbol{a}_i$:

$$\mathcal{I}_i = \left(\boldsymbol{\nabla}\langle\hat{\boldsymbol{\mu}}\rangle \cdot \boldsymbol{a}_i\right)^2 . \tag{S1}$$

Fig. S1 shows the $|a_c|^2$ values and the harmonic intensities $\mathcal{I}$ of the relevant transitions as a function of the coupling strength λ_m for optimized H_2O-cavity systems.

In the case of a single cavity mode coupled to H_2O, only three transitions are relevant to explain the spectral features in the vicinity of $1744\,\mathrm{cm}^{-1}$. The main transition ν_2 shown as a green dashed line in Fig. S1 a) and b) corresponds to the molecular bending mode. It does not hybridize with the photon mode and its intensity is constant with increasing λ_m.

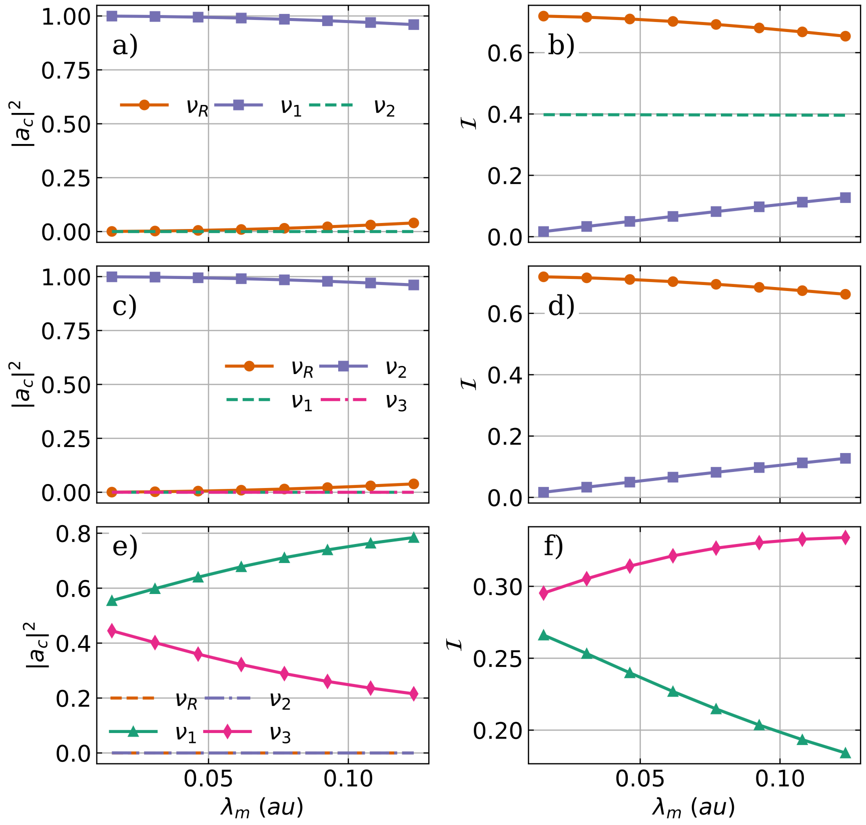

Figure S1: Relevant $|a_c|^2$ values and harmonic intensities $\mathcal{I}$ as a function of the coupling strength λ_m for optimized H_2O structures coupled to a single cavity mode a) and b) as well as to two orthogonal cavity modes c), d), e) and f). The values for the cavity mode with the polarization axis $\boldsymbol{e}_2$ orthogonal to the dipole moment are given in c) and d) and for the one with the polarization axis $\boldsymbol{e}_1$ parallel to the dipole moment in e) and f). The cavity frequency ω_c is $1744\,\mathrm{cm}^{-1}$ and the cavity coupling λ_m increases from $0.015\,\mathrm{au}$ to $0.123\,\mathrm{au}$.

In contrast, ν_1, shown as purple in Fig. S1 a) and b), is purely photonic at low coupling and has an intensity close to zero. With increasing coupling strength, ν_1 begins to weakly couple to a rotational mode (ν_R orange line). As a consequence, ν_R becomes slightly photonic and at the same time ν_1 gains intensity.

In the case of H_2O coupled to two orthogonal cavity modes, we divide the discussion into two parts: First, the photon mode orthogonal to the dipole moment shown in Fig. S1 c) and d) and second, the one parallel to the dipole mode shown in Fig. S1 e) and f). For the orthogonal cavity mode, the picture is almost identical to the single-mode case. The photon mode transition ν_2 is almost decoupled and dark but starts to weakly couple to the

rotational mode transition ν_R with increasing coupling strength. In contrast, in the case of the parallel cavity mode, the transitions ν_1 and ν_3 are clearly hybridized already for low coupling strengths and stay so for increasing λ_m.

In Fig. S2 we show the $|a_c|^2$ values of the four relevant transitions for the optimized H_2O_2 molecule coupled to a single cavity mode as a function of the coupling strength λ_m. The asymmetric bending mode transition ν_4 (pink) and the cavity photon mode transition

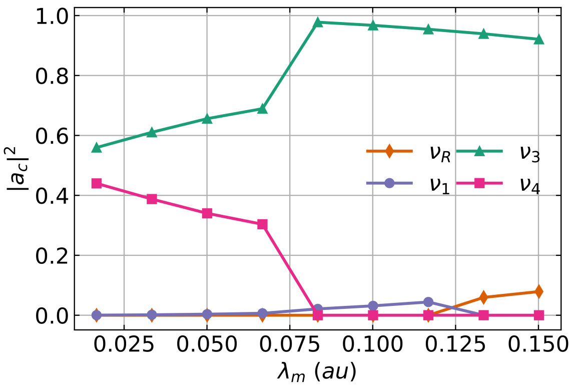

Figure S2: $|a_c|^2$ values of the four relevant transitions for the optimized H_2O_2 molecule coupled to a single cavity mode as a function of the coupling strength λ_m. The cavity frequency ω_c is 1491 cm^{-1} and the cavity coupling λ_m increases from 0.015 au to 0.150 au.

ν_3 (green) are clearly hybridized for low coupling strengths, smaller than 0.075 au. Due to geometrical changes, the cavity photon mode transition ν_3 decouples from ν_4 for larger λ_m and starts to interact weakly with the H_2O_2 twisting mode (ν_1 purple). For even higher coupling strengths and close to planarization, ν_3 is weakly coupled to a rotational mode (ν_R orange), similar to the H_2O case.

The $|a_c|^2$ values of the four relevant transitions for the optimized H_2O_2 molecule coupled to two orthogonal cavity modes are depicted in Fig. S3.

For the orthogonal cavity mode Fig. S3 a), the corresponding photon mode transition ν_4 (green) starts almost decoupled and dark, but starts to couple weakly to the H_2O_2 twisting mode (ν_1 orange) with increasing coupling strength. In contrast, in the case of the parallel cavity mode Fig. S3 b), the transitions ν_3 and ν_5 are clearly hybridized already at low coupling strengths and stay so for increasing λ_m.

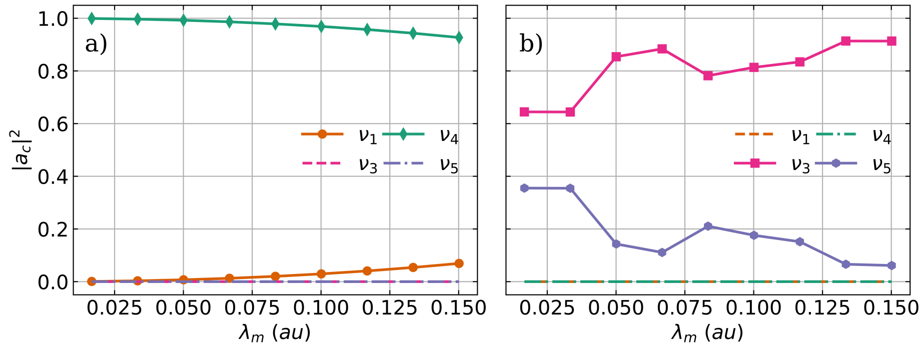

Figure S3: $|a_c|^2$ values of the four relevant transitions for the optimized H_2O_2 molecule coupled to two orthogonal cavity modes as a function of the coupling strength λ_m. a) For the cavity mode with the polarization axis $\boldsymbol{e}_2$ orthogonal to the dipole moment and b) for the one with the polarization axis $\boldsymbol{e}_1$ parallel to the dipole moment. The cavity frequency ω_c is $1491\,\mathrm{cm}^{-1}$ and the cavity coupling λ_m increases from 0.015 au to 0.150 au.

S2 Benchmark Optimization Methods and Parameters

We benchmark the performance of various algorithms for the optimization of molecules coupled to cavity photon modes in the CBOA representation. As a test case, we optimize a H_2O molecule coupled to a cavity. For the case of a single cavity mode, $\boldsymbol{e}$ is neither parallel nor orthogonal to the molecular plane. In the case of two modes, $\boldsymbol{e}_1$ is in the molecular plane and $\boldsymbol{e}_2$ is aligned with the normal vector of the molecular plane. The simplest algorithm used is the Steepest Descent (SD) method, which requires only the gradient in each optimization step. The most computationally intensive is the Newton–Raphson (NR) method, which requires the exact Hessian matrix in each step. The Broyden–Fletcher–Goldfarb–Shanno (BFGS) algorithm offers a middle ground between SD and NR in terms of computational complexity. It uses an updating scheme that approximates the Hessian matrix at each point n using the gradient, the displacement, and the Hessian matrix at step $n-1$. For the simplest version of the BFGS method, the identity matrix is used as an approximate Hessian at $n = 0$. To improve the performance, the exact Hessian can be used in the first step and during the optimization the exact Hessian can be recomputed after a certain number of steps. We have included an augmented Hessian method for each optimization routine in which we use the

exact Hessian to ensure that the Hessian matrix is positively defined.

$$\boldsymbol{x}_{n+1} = \boldsymbol{x}_n - \left(\boldsymbol{H} - (\min(\epsilon) + \gamma))\mathbf{1}\right)^{-1} \boldsymbol{g}\,. \tag{S2}$$

Here, $\min(\epsilon)$ is the smallest eigenvalue of the Hessian matrix and γ is a small number to prevent the augmented Hessian matrix from being non-invertible. The augmentation is necessary to avoid inadvertently optimizing a transition state or a higher-order saddle point.

Fig. S4 shows the convergence behavior for different optimization algorithms.

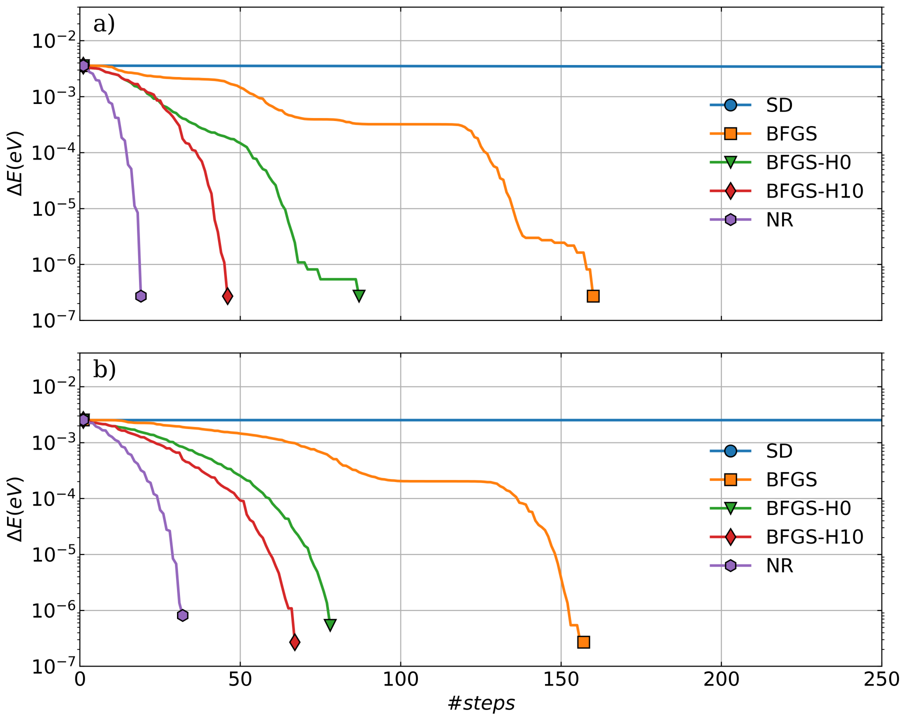

Figure S4: Energy convergence for the optimization of a single H_2O molecule coupled to a) one cavity photon mode or b) two cavity photon modes for $\omega_m = 1744.0\,\mathrm{cm}^{-1}$ and $\lambda_m = 0.031$ au using different optimization routines. The different optimization routines are color-coded. The different versions of the BFGS method are labeled as follows: BFGS started with the identity matrix and no Hessian was computed, BFGS-H0 started with the exact Hessian, and BFGS-H10 started with the exact Hessian and recomputed it every 10th step. The starting point and the converged end point are marked.

The SD method does not converge within the maximum number of 250 steps. The NR method and all versions of the BFGS method converge to the same minimum structure. As

expected, the NR method shows the fastest convergence, while the simplest BFGS version needs about three times more optimization steps. Including Hessian information in the BFGS algorithm significantly speeds up convergence, especially when the exact Hessian is recomputed during optimization. In Fig. S5 the energy convergence for different intervals for the reactivation of the Hessian is shown.

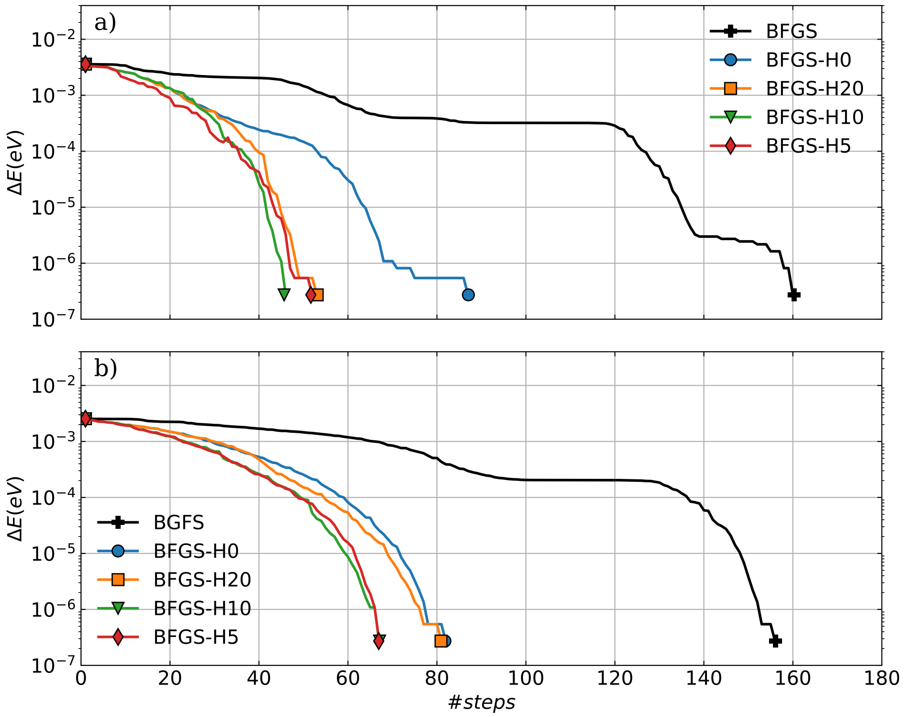

Figure S5: Energy convergence for the optimization of a single H_2O molecule coupled to a) one cavity photon mode or b) two cavity photon modes for $\omega_m = 1744.0\,\mathrm{cm}^{-1}$ and $\lambda_m = 0.031\,\mathrm{au}$ using different versions of the BFGS routine. Different intervals for recalculation of the exact Hessian are color-coded. TheBFGS results without the exact Hessian are shown in black. The starting point and the converged end point are marked.

For the case of H_2O coupled to a single cavity mode (Fig. S6 a)), recalculating the exact Hessian during optimization significantly speeds up convergence. However, changing the interval from every 20th step to every 5th step shows only a small improvement. This finding also holds for the case of two orthogonal cavity modes, shown in Fig. S6 b).

To improve convergence and overall stability, we implement a trust-radius approach combined with a backtracking line search in our optimization routines. The trust-radius approach

sets an upper bound R for the step size, while the backtracking line search further reduces the step size if the energy increases along the step. The latter comes with a coast of additional single-point calculations during each optimization step. The energy convergence for different trust radii R is shown in Fig. S6 and for different number of iterations of the backtracking line search is shown in Fig. S7.

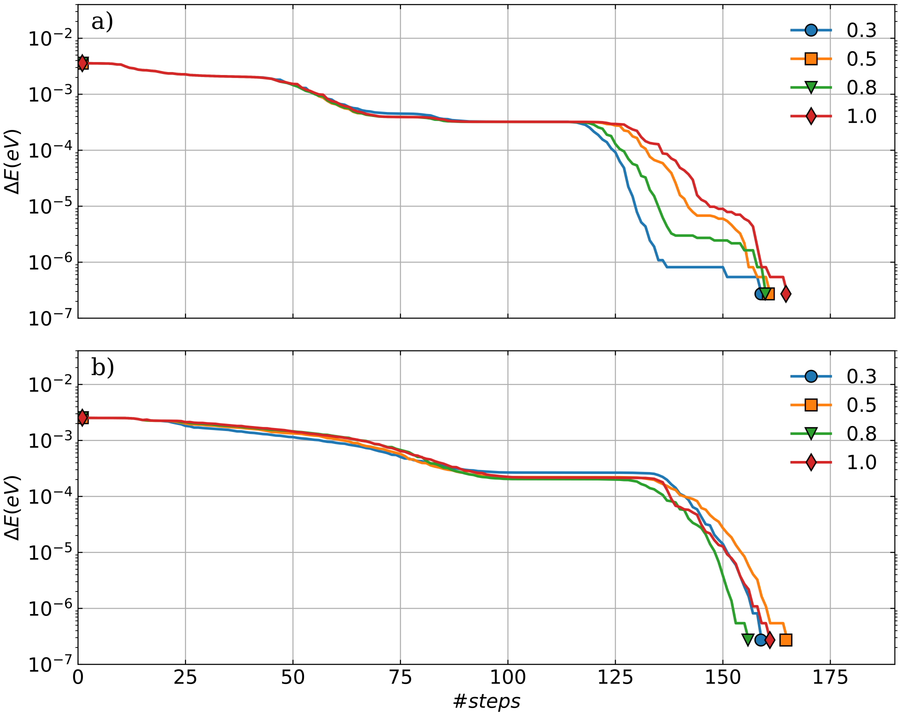

Figure S6: Energy convergence for the optimization of a single H_2O molecule coupled to a) one cavity photon mode or b) two cavity photon modes for $\omega_m = 1744.0\,\mathrm{cm}^{-1}$ and $\lambda_m = 0.031\,\mathrm{au}$ using the BFGS routine recalculating the Hessian in every 10th step and 5 steps in the backtracking line search. Different values for the trust radius R are color-coded. The starting point and the converged end point are marked.

The chosen size of the trust radius R has only a small influence on convergence, and a value of $0.8\,\mathrm{au}$ shows the best results (green line in Fig. S6). In contrast, the number of iterations of the backtracking line search has an impact on both stability and convergence. With more steps in the line search, the average step size is getting smaller, and therefore more steps are needed. However, without line search (blue line in Fig. S7), the energy change is highly fluctuating, making optimization more unstable. The best results in both stability

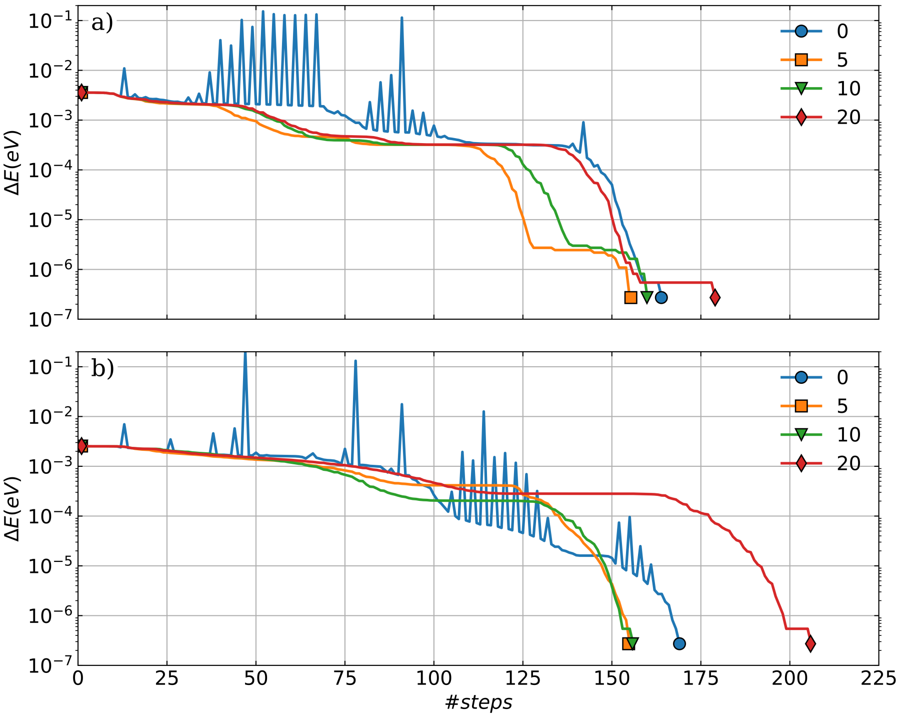

Figure S7: Energy convergence for the optimization of a single H_2O molecule coupled to a) one cavity photon mode or b) two cavity photon modes for $\omega_m = 1744.0\,\mathrm{cm}^{-1}$ and $\lambda_m = 0.031\,\mathrm{au}$ using the BFGS routine recalculating the Hessian in every 10th step and a trust radius of $0.8\,\mathrm{au}$. Different numbers of steps in the backtracking line search are color-coded. The starting point and the converged end point are marked.

and convergence are achieved for 5 steps in the backtracking line search (orange line in Fig. S7)

S3 Optimized geometries

S3.1 Optimized H_2O coupled to a single cavity mode

All optimizations were performed using the BFGS method, starting from the exact Hessian matrix in the first step and recalculating it after every 10th step. The minima found were verified to have no imaginary frequency.

Table 1: $\lambda_m = 0.015$ au and $q_1 = 0.0000$ au.

O	0.0000	−0.0282	−0.1101
H	0.0000	−0.6170	0.6273
H	0.0000	0.8425	0.2536

Table 2: $\lambda_m = 0.031$ au and $q_1 = 0.0000$ au.

O	0.0000	−0.0260	−0.1107
H	0.0000	−0.6294	0.6146
H	0.0000	0.8370	−0.2707

Table 3: $\lambda_m = 0.046$ au and $q_1 = 0.0000$ au.

O	0.0000	−0.0252	−0.1108
H	0.0000	−0.6335	0.6107
H	0.0000	0.8349	0.2766

Table 4: $\lambda_m = 0.062$ au and $q_1 = 0.0000$ au.

O	0.0000	−0.0070	−0.1134
H	0.0000	−0.7233	0.5000
H	0.0000	0.7791	0.4075

Table 5: $\lambda_m = 0.077$ au and $q_1 = 0.0000$ au.

O	0.0000	−0.2666	−0.1105
H	0.0000	−0.6247	0.6182
H	0.0000	0.8379	0.2655

Table 6: $\lambda_m = 0.093$ au and $q_1 = 0.0000$ au.

O	0.0000	−0.0036	0.1136
H	0.0000	−0.7372	−0.4779
H	0.0000	0.7658	−0.4305

Table 7: $\lambda_m = 0.101$ au and $q_1 = 0.0000$ au.

O	0.0000	0.0099	0.1132
H	0.0000	−0.7880	−0.3874
H	0.0000	0.7091	−0.5179

Table 8: $\lambda_m = 0.123$ au and $q_1 = 0.0000$ au.

O	0.0000	0.0027	−0.1135
H	0.0000	−0.7614	0.4363
H	0.0000	0.7398	0.4720

S3.2 Optimized H_2O coupled to two orthogonal cavity modes

All optimizations were performed using the BFGS method, starting from the exact Hessian matrix in the first step and recalculating it after every 10th step. The minima found were verified to have no imaginary frequency.

Table 9: $\lambda_m = 0.015\,\text{au}$, $q_1 = -1.5005\,\text{au}$ and $q_2 = 0.0000\,\text{au}$.

O	−0.1136	0.0000	0.0000
H	0.4542	0.0000	0.7535
H	0.4542	0.0000	−0.7535

Table 10: $\lambda_m = 0.031\,\text{au}$, $q_1 = -3.0012\,\text{au}$ and $q_2 = 0.0000\,\text{au}$.

O	−0.1135	0.0000	0.0000
H	0.4536	0.0000	0.7535
H	0.4536	0.0000	−0.7535

Table 11: $\lambda_m = 0.046\,\text{au}$, $q_1 = -4.5021\,\text{au}$ and $q_2 = 0.0000\,\text{au}$.

O	−0.1132	0.0000	0.0000
H	0.4526	0.0000	0.7536
H	0.4526	0.0000	−0.7536

Table 12: $\lambda_m = 0.062\,\text{au}$, $q_1 = -6.0033\,\text{au}$ and $q_2 = 0.0000\,\text{au}$.

O	−0.1129	0.0000	0.0000
H	0.4513	0.0000	0.7537
H	0.4513	0.0000	−0.7537

Table 13: $\lambda_m = 0.077\,\text{au}$, $q_1 = -7.5050\,\text{au}$ and $q_2 = 0.0000\,\text{au}$.

O	−0.1125	0.0000	0.0000
H	0.4497	0.0000	0.7538
H	0.4497	0.0000	−0.7538

Table 14: $\lambda_m = 0.093\,\text{au}$, $q_1 = -9.0068\,\text{au}$ and $q_2 = 0.0000\,\text{au}$.

O	−0.1120	0.0000	0.0000
H	0.4480	0.0000	0.7538
H	0.4480	0.0000	−0.7538

Table 15: $\lambda_m = 0.101\,\text{au}$, $q_1 = -10.5084\,\text{au}$ and $q_2 = 0.0000\,\text{au}$.

O	−0.1114	0.0000	0.0000
H	0.4453	0.0000	0.7542
H	0.4460	0.0000	−0.7542

Table 16: $\lambda_m = 0.123\,\text{au}$, $q_1 = -12.0095\,\text{au}$ and $q_2 = 0.0000\,\text{au}$.

O	−0.1108	0.0000	0.0000
H	0.4430	0.0000	0.7543
H	0.4430	0.0000	−0.7543

S3.3 Optimized H_2O_2 coupled to a single cavity mode

All optimizations were performed using the BFGS method, starting from the exact Hessian matrix in the first step. The minima found were verified to have no imaginary frequency.

Table 17: $\lambda_m = 0.017$ au and $q_1 = 1.8231$ au.

O	−0.1693	−0.6737	0.0575
H	0.5197	−1.0623	−0.4600
O	0.1693	0.6737	0.0575
H	−0.5197	1.0623	−0.4600

Table 18: $\lambda_m = 0.033$ au and $q_1 = 3.5933$ au.

O	−0.1066	−0.6864	0.0566
H	0.6204	−1.0101	−0.4529
O	0.1066	0.6864	0.0566
H	−0.6204	1.0101	−0.4529

Table 19: $\lambda_m = 0.050$ au and $q_1 = 5.2569$ au.

O	−0.1731	−0.6727	0.0551
H	0.5276	−1.0669	−0.4406
O	0.1731	0.6727	0.0551
H	−0.5276	1.0669	−0.4406

Table 20: $\lambda_m = 0.067\,\text{au}$ and $q_1 = 6.7444\,\text{au}$.

O	−0.1766	−0.6719	0.0529
H	0.5342	−1.0711	−0.4229
O	0.1766	0.6719	0.0529
H	−0.5342	1.0711	−0.4229

Table 21: $\lambda_m = 0.083\,\text{au}$ and $q_1 = 8.7840\,\text{au}$.

O	−0.1869	−0.6693	0.0456
H	0.5535	−1.0840	−0.3644
O	0.1869	0.6693	0.0456
H	−0.5535	1.0840	−0.3644

Table 22: $\lambda_m = 0.100\,\text{au}$ and $q_1 = 8.9450\,\text{au}$.

O	−0.1944	−0.6675	0.0396
H	0.5662	−1.0934	−0.3169
O	0.1944	0.6675	0.0396
H	−0.5662	1.0934	−0.3169

Table 23: $\lambda_m = 0.117\,\text{au}$ and $q_1 = 7.7993\,\text{au}$.

O	−0.2030	−0.6656	0.0301
H	0.5841	−1.1051	−0.2407
O	0.2030	0.6656	0.0301
H	−0.5841	1.1051	−0.2407

Table 24: $\lambda_m = 0.133\,\text{au}$ and $q_1 = 0.0007\,\text{au}$.

O	−0.2124	−0.6638	0.0251
H	0.6112	−1.1193	−0.1507
O	0.2124	0.6638	0.0251
H	−0.6112	1.1193	−0.1507

Table 25: $\lambda_m = 0.150\,\text{au}$ and $q_1 = 0.0000\,\text{au}$.

O	−0.2130	−0.6631	0.0000
H	0.6097	−1.1189	0.0000
O	0.2130	0.6097	0.0000
H	−0.5197	1.1189	0.0000

S3.4 Optimized H_2O_2 coupled to two orthogonal cavity modes

All optimizations were performed using the BFGS method, starting from the exact Hessian matrix in the first step. The minima found were verified to have no imaginary frequency.

Table 26: $\lambda_m = 0.017\,\text{au}$, $q_1 = 1.8296\,\text{au}$ and $q_2 = 0.0000\,\text{au}$.

O	−0.2056	−0.6635	0.0577
H	0.4597	−1.0887	−0.4618
O	0.2056	0.6635	0.0577
H	−0.4597	1.0887	−0.4618

Table 27: $\lambda_m = 0.033\,\text{au}$, $q_1 = 3.6526\,\text{au}$ and $q_2 = 0.0000\,\text{au}$.

O	−0.2060	−0.6630	0.0575
H	0.4591	−1.0894	−0.4602
O	0.2060	0.6630	0.0575
H	−0.4591	1.0894	−0.4602

Table 28: $\lambda_m = 0.050\,\text{au}$, $q_1 = 5.4627\,\text{au}$ and $q_2 = 0.0000\,\text{au}$.

O	−0.2067	−0.6623	0.0572
H	0.4581	−1.0904	−0.4575
O	0.2067	0.6623	0.0572
H	−0.4581	1.0904	−0.4575

Table 29: $\lambda_m = 0.067\,\text{au}$, $q_1 = 7.2555\,\text{au}$ and $q_2 = 0.0000\,\text{au}$.

O	−0.2076	−0.6612	0.0567
H	0.4569	−1.0920	−0.4539
O	0.2076	0.6612	0.0567
H	−0.4569	1.0920	−0.4539

Table 30: $\lambda_m = 0.083\,\text{au}$, $q_1 = 9.0194\,\text{au}$ and $q_2 = 0.0000\,\text{au}$.

O	−0.2085	−0.6600	0.0562
H	0.4555	−1.0938	−0.4493
O	0.2085	0.6600	0.0562
H	−0.4555	1.0938	−0.4493

Table 31: $\lambda_m = 0.100\,\text{au}$, $q_1 = 10.7484\,\text{au}$ and $q_2 = 0.0000\,\text{au}$.

O	−0.2097	−0.6585	0.0555
H	0.4538	−1.0961	−0.4437
O	0.2097	0.6585	0.0555
H	−0.4538	1.0961	−0.4437

Table 32: $\lambda_m = 0.117\,\text{au}$, $q_1 = 12.4338\,\text{au}$ and $q_2 = 0.0000\,\text{au}$.

O	−0.2111	−0.6568	0.0547
H	0.4519	−1.0988	−0.4372
O	0.2111	0.6568	0.0547
H	−0.4519	1.0988	−0.4372

Table 33: $\lambda_m = 0.133\,\text{au}$, $q_1 = 14.0671\,\text{au}$ and $q_2 = 0.0000\,\text{au}$.

O	−0.2126	−0.6548	0.0537
H	0.4495	−1.1018	−0.4300
O	0.2126	0.6548	0.0537
H	−0.4495	1.1018	−0.4300

Table 34: $\lambda_m = 0.150\,\text{au}$, $q_1 = 15.6389\,\text{au}$ and $q_2 = 0.0000\,\text{au}$.

O	−0.2144	−0.6526	0.0527
H	0.4468	−1.1053	−0.4219
O	0.2144	0.6526	0.0527
H	−0.4468	1.1053	−0.4219

References

(1) Schnappinger, T.; Kowalewski, M. Ab Initio Vibro-Polaritonic Spectra in Strongly Coupled Cavity-Molecule Systems. *J. Chem. Theory Comput.* **2023**, *19*, 9278–9289.

(2) Rokaj, V.; Welakuh, D. M.; Ruggenthaler, M.; Rubio, A. Light–matter interaction in the long-wavelength limit: no ground-state without dipole self-energy. *J. Phys. B At. Mol. Opt. Phys.* **2018**, *51*, 034005.

(3) Schäfer, C.; Ruggenthaler, M.; Rokaj, V.; Rubio, A. Relevance of the Quadratic Diamagnetic and Self-Polarization Terms in Cavity Quantum Electrodynamics. *ACS Photonics* **2020**, *7*, 975–990.

(4) Welakuh, D. M.; Rokaj, V.; Ruggenthaler, M.; Rubio, A. Non-perturbative mass renormalization effects in non-relativistic quantum electrodynamics. **2023**,

(5) Hopfield, J. J. Theory of the Contribution of Excitons to the Complex Dielectric Constant of Crystals. *Phys. Rev.* **1958**, *112*, 1555–1567.
